# Studies of insulin and proinsulin in pancreas and serum support the existence of aetiopathological endotypes of type 1 diabetes associated with age at diagnosis

**DOI:** 10.1007/s00125-020-05115-6

**Published:** 2020-03-15

**Authors:** Pia Leete, Richard A. Oram, Timothy J. McDonald, Beverley M. Shields, Clemens Ziller, TIGI study team, Bart O. Roep, Timothy I. Tree, Kashyap Patel, Suzy Hammersley, Robert Bolt, Anita V. Hill, Andrew T. Hattersley, Sarah J. Richardson, Noel G. Morgan

**Affiliations:** Institute of Biomedical and Clinical Science, University of Exeter Medical School, RILD Building, Barrack Road, Exeter, EX2 5DW UK

**Keywords:** CD20^+^ cells, CD8^+^ cells, C-peptide, Immunophenotype, Insulitis, Islets of Langerhans

## Abstract

**Aims/hypothesis:**

It is unclear whether type 1 diabetes is a single disease or if endotypes exist. Our aim was to use a unique collection of pancreas samples recovered soon after disease onset to resolve this issue.

**Methods:**

Immunohistological analysis was used to determine the distribution of proinsulin and insulin in the islets of pancreas samples recovered soon after type 1 diabetes onset (<2 years) from young people diagnosed at age <7 years, 7–12 years and ≥13 years. The patterns were correlated with the insulitis profiles in the inflamed islets of the same groups of individuals. C-peptide levels and the proinsulin:C-peptide ratio were measured in the circulation of a cohort of living patients with longer duration of disease but who were diagnosed in these same age ranges.

**Results:**

Distinct patterns of proinsulin localisation were seen in the islets of people with recent-onset type 1 diabetes, which differed markedly between children diagnosed at <7 years and those diagnosed at ≥13 years. Proinsulin processing was aberrant in most residual insulin-containing islets of the younger group but this was much less evident in the group ≥13 years (*p* < 0.0001). Among all individuals (including children in the middle [7–12 years] range) aberrant proinsulin processing correlated with the assigned immune cell profiles defined by analysis of the lymphocyte composition of islet infiltrates. C-peptide levels were much lower in individuals diagnosed at <7 years than in those diagnosed at ≥13 years (median <3 pmol/l, IQR <3 to <3 vs 34.5 pmol/l, IQR <3–151; *p* < 0.0001), while the median proinsulin:C-peptide ratio was increased in those with age of onset <7 years compared with people diagnosed aged ≥13 years (0.18, IQR 0.10–0.31) vs 0.01, IQR 0.009–0.10 pmol/l; *p* < 0.0001).

**Conclusions/interpretation:**

Among those with type 1 diabetes diagnosed under the age of 30 years, there are histologically distinct endotypes that correlate with age at diagnosis. Recognition of such differences should inform the design of future immunotherapeutic interventions designed to arrest disease progression.

**Electronic supplementary material:**

The online version of this article (10.1007/s00125-020-05115-6) contains peer-reviewed but unedited supplementary material, which is available to authorised users.

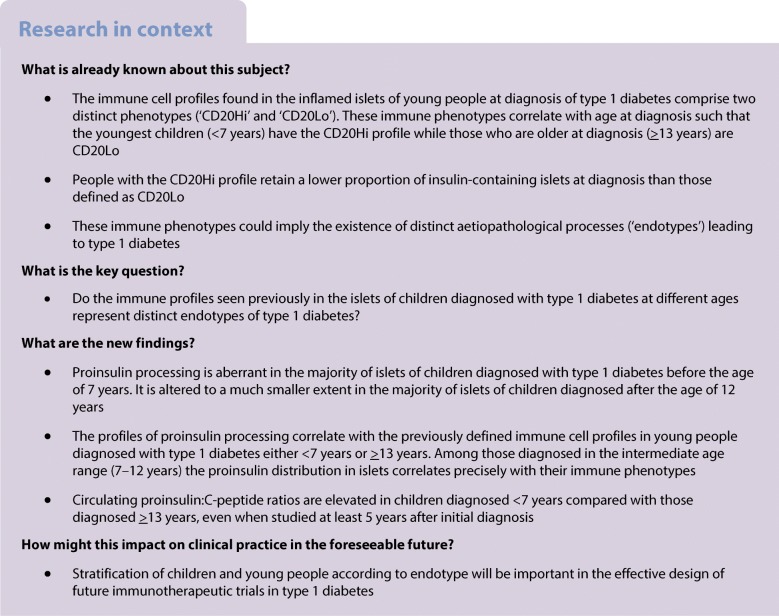



## Introduction

Type 1 diabetes is an autoimmune disease caused by destruction of pancreatic beta cells leading to severe insulin deficiency. Most attempts to prevent, slow or reverse the disease process have been unsuccessful [1–3], although recent clinical trials undertaken with the humanised anti-CD3 reagent teplizumab suggest that targeted interventions can be effective [4]. One obstacle to the development of still more effective therapeutic approaches is a continuing incomplete understanding of the aetiopathology of the disease at the level of the pancreas. Therefore, to address this, we have made use of both the Network of Pancreatic Organ Donors (nPOD) biobank of organ donor pancreas samples collected in the USA and a separate, unique, collection of pancreases recovered from young people close to the clinical onset of type 1 diabetes from the UK [5, 6]. In particular, we have adopted a histological approach to consider an important question raised by emerging data (reviewed recently by Battaglia et al [7]) which imply that type 1 diabetes may not represent a single disease but that distinct endotypes exist. This concept is of critical importance since, if verified, it suggests that different immunotherapeutic approaches will be required to achieve successful intervention in specific groups of patients.

Previously, we described the existence of two discrete histological profiles of pancreatic insulitis that associate strongly with age at diagnosis of type 1 diabetes [8, 9]. In principle, these data are consistent with the existence of disease endotypes but such a conclusion must be substantiated by the assessment of additional variables. Therefore, we have now adopted an entirely independent approach to evaluate more fully whether disease endotypes can be distinguished at the level of the human pancreas. To achieve this, we have studied the processing of proinsulin in the islets of children and young people with recent-onset type 1 diabetes since elevations in circulating proinsulin have been correlated with age at diagnosis in individuals recently diagnosed with type 1 diabetes [10–12]. More specifically, we have assessed the intracellular distribution of proinsulin and insulin in the islets of individuals across three age ranges (<7 years, 7–12 years, >13 years). This analysis was undertaken in a blinded manner and the results then correlated with the profile of infiltrating immune cells in the islets of these same individuals. We have also examined whether the circulating proinsulin:C-peptide ratio measured in individuals with longer-standing type 1 diabetes and diagnosed across these same age ranges mirrors the histological changes seen in the pancreas.

## Methods

### Pancreas samples for histopathological analysis

Pancreatic samples for study of immune cell infiltration were described previously [8, 13] and details are given in electronic supplementary material (ESM) Tables 1–3. Pancreatic samples for study of proinsulin and insulin co-localisation were obtained from within the Exeter Archival Diabetes Biobank (EADB) or the nPOD collection. With one exception, patients with recent-onset type 1 diabetes in whom the localisation of proinsulin and insulin were studied had been diagnosed under 20 years of age (median 10.5 years, IQR 6.25–18.00 years). A total of 19 patients with recent-onset (<2 years’ duration) type 1 diabetes (ESM Tables 1 and 4) and 13 with longer-duration (>5 years) disease were studied (ESM Tables 3 and 5) with full ethics approval (West of Scotland Research Ethics Committee, reference: 15/WS/0258).

### Staining of pancreatic samples

#### Immunostaining for insulin and proinsulin

Pancreas sections were immunostained for insulin and proinsulin using standard immunofluorescent protocols. Staining was achieved with validated antibodies that were highly selective for proinsulin or mature insulin (ESM Table 6). Insulin-containing islets (ICIs, *n* = 488) were studied across the 32 samples to establish the co-localisation profiles. The sections were imaged via a Leica DMi8 confocal microscope (Leica Microsystems [UK], Milton Keynes, UK) and the distribution of proinsulin and insulin examined in multiple islets using ImageJ (JACoP plugin [14]; ImageJ, Bethesda, MD, USA). For analysis of proinsulin:insulin localisation profiles in the islets of each individual, images were analysed in a blinded manner among individuals across the age ranges studied and samples were selected arbitrarily in random order for analysis. The Manders overlap coefficient (MOC) is the method of choice for establishing the extent of co-localisation of two proteins and was calculated as an index of the extent of co-localisation of proinsulin with insulin [15].

#### Insulitis profiling

Sections from each case were also immunostained to detect CD4^+^, CD8^+^ and CD20^+^ cells using validated antibodies, as described previously [8, 9]. Primary antibodies are listed in ESM Table 6 and the immunohistochemical analysis was performed using Agilent/DAKO Envision reagents (Agilent Technologies, Cheadle, Cheshire, UK) or Alexafluor fluorophores (ThermoFisher, Loughborough, UK) with or without the Tyramide SuperBoost (ThermoFisher) protocol. Immunophenotyping was achieved as described previously [8].

### C-peptide, proinsulin and antibody measurement in living individuals with long-duration disease

We examined 171 individuals with long-duration type 1 diabetes (>5 years) to assess their serum C-peptide, proinsulin and proinsulin:C-peptide ratio. We recruited these participants as part of the Type 1 diabetes, Immunology, Genetics and endogenous Insulin production (TIGI) study, designed to study associations of persistent C-peptide in type 1 diabetes, and their clinical details are given in ESM Table 7. All participants were diagnosed under 30 years, had a clinical diagnosis of type 1 diabetes and were insulin treated from diagnosis. Patients were included if their original diagnosis was made at ages <7 years (*n* = 87) or ≥13 years (*n* = 84). We measured stimulated serum proinsulin and C-peptide 90 min after a mixed-meal tolerance test. We compared proinsulin levels and proinsulin:C-peptide ratio in people with long-duration type 1 diabetes with routine clinical samples from 39 people without diabetes (age range 3–25 years). All participants provided informed consent and the National Research Ethics Service Committee South West approved the TIGI study (13/SW/0312).

### Biochemical analyses

Serum C-peptide was analysed using a direct electrochemiluminescence immunoassay on the 602 module of the COBAS 8000 platform (Roche Diagnostics, Mannheim, Germany). The limit of detection was 3.3 pmol/l and intra assay CV <4.7%. Proinsulin was measured using the TECO Medical Intact Proinsulin ELISA kit (TECO, Sissach, Switzerland) on the Dynex DS2 analyser (Dynex Technologies, Chantilly, VA, USA). Limit of detection was 0.3 pmol/l; intra assay precision <7.5% (full details are provided in the ESM Validation Method). C-peptide and proinsulin results below the limit of the assay were recorded as 2.9 pmol/l and 0.29 pmol/l, respectively. Proinsulin:C-peptide ratio was calculated only for those who had at least one detectable value. The assumption that the undetectable analyte (most commonly C-peptide) was just below the lower limit of assay detection provided a conservative estimate of proinsulin:C-peptide ratio.

### Statistical analysis

The distribution of proinsulin and insulin in the pancreatic samples from the different age groups at diagnosis was compared using the Kolmogorov–Smirnov test. In samples from living individuals, differences in C-peptide and proinsulin:C-peptide ratio between subgroups were compared using the Mann–Whitney *U* test. Data are presented in dot plot format showing individual values with median and interquartile ranges. Groups were considered statistically different where the *p* value was <0.05.

## Results

### Examination of proinsulin distribution in the islets of children diagnosed with type 1 diabetes at different ages

**Fig. 1:**
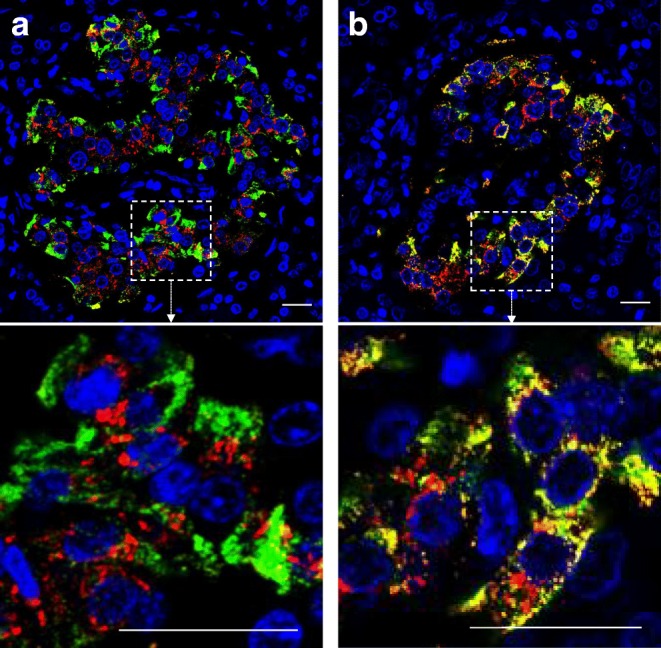
Fluorescence micrographs showing (**a**) Islet in which proinsulin and insulin are segregated. Enlarged region (dotted square in upper panel) is shown in the lower panel. (**b**) Islet with aberrant proinsulin processing. Enlarged region (dotted square in upper) is shown in lower panel. Green, insulin; red, proinsulin; yellow, co-localisation of the antigens. Scale bar, 20 μm

When analysed in a blinded manner, two strikingly different patterns of proinsulin immunostaining were seen in the pancreases of children and young people recently diagnosed with type 1 diabetes. In one group, proinsulin was predominantly distributed within a perinuclear compartment that was largely devoid of insulin immunostaining, whereas mature insulin was localised more widely within the cytoplasm (Fig. 1a). Accordingly, the median MOC was <0.5. In a second group, the immunostaining pattern was strikingly different in that proinsulin and insulin were unexpectedly co-localised throughout the cytoplasm of the beta cells (Fig. 1b). This was reflected in a median MOC for co-localisation of the two antigens of >0.5.

**Fig. 2:**
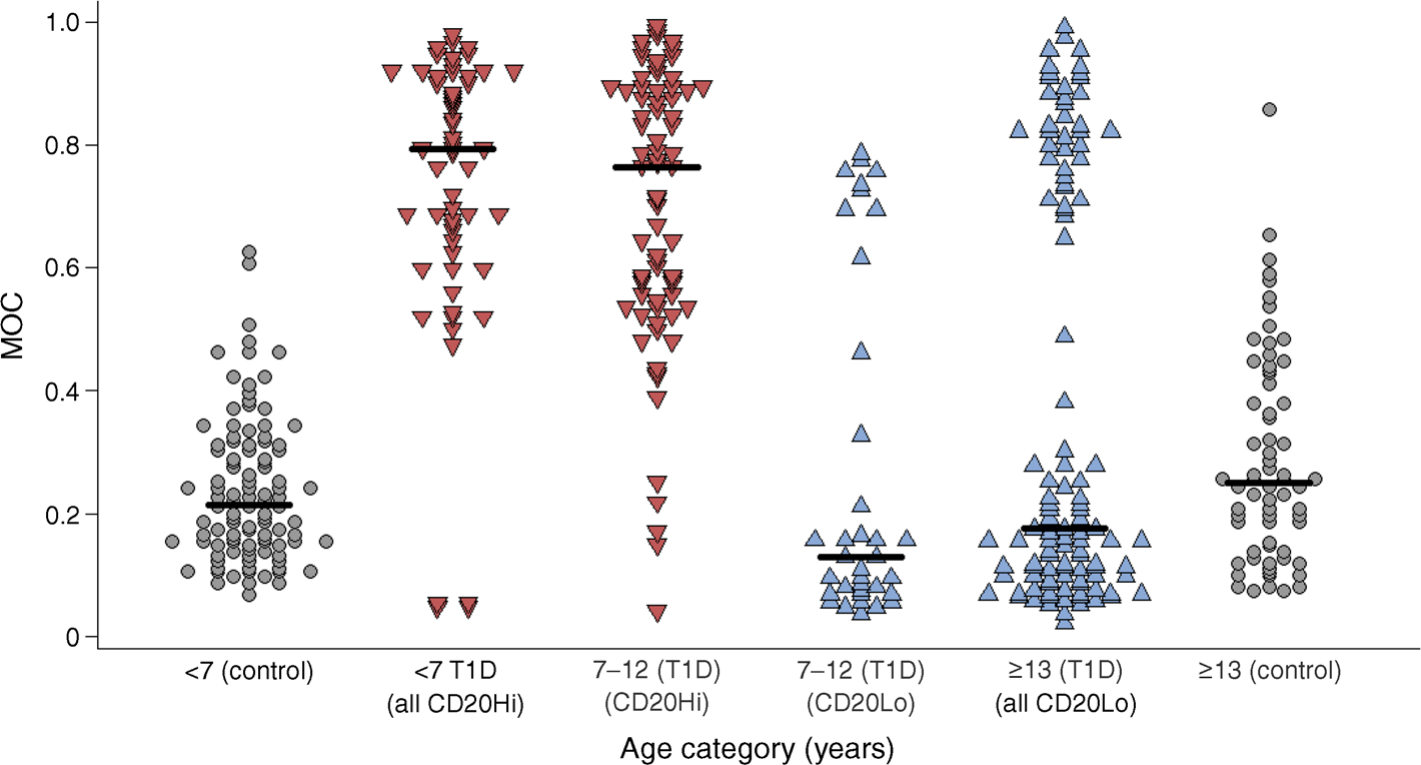
Dot plot showing distribution of the MOC (a measure of proinsulin–insulin co-localisation) in individual islets from pancreas samples obtained within 2 years of diagnosis of type 1 diabetes for individuals with disease onset at <7 years, 7–12 years and ≥13 years. Individuals in each age group were separated into CD20Hi (red) and CD20Lo (blue) subgroups, respectively. Data from control individuals without diabetes are presented as grey circles for those aged <7 years (far left) or ≥13 years (far right). Black horizontal bars represent median values for each group

Examination of the demographic features of the people falling within the histologically distinct groups revealed that the children in whom most ICIs displayed high proinsulin–insulin co-localisation (MOC >0.5) were in the younger age group (under 13 years). More specifically, all five individuals diagnosed at <7 years had high proinsulin–insulin co-localisation (MOC: median 0.794, IQR 0.625–0.913) whereas six of seven individuals diagnosed ≥13 years had low proinsulin–insulin co-localisation (MOC: median 0.175, IQR 0.10–0.35; *p* < 0.0001) (Fig. 2). This age distribution is strongly reminiscent of the age profiles defining the two immune cell phenotypes reported previously in the inflamed islets of young people [8]. Hence, in children aged <7 years or ≥13 years at diagnosis, there was a strong correlation between the insulitis profiles defined previously as ‘CD20Hi’ or ‘CD20Lo’ [8] and the patterns of proinsulin immunostaining (Fig. 2). Children diagnosed at <7 years and designated as ‘CD20Hi’ displayed unexpectedly high levels of proinsulin–insulin co-localisation. By contrast, those diagnosed ≥13 years (‘CD20Lo’) had minimal proinsulin:insulin co-localisation in most islets (Fig. 2). Study of the proinsulin–insulin profiles seen in individuals of equivalent ages (either <7 years or ≥13 years) who did not have type 1 diabetes, revealed that very few islets were present in which proinsulin co-localised with insulin (Fig. 2).

**Fig. 3:**
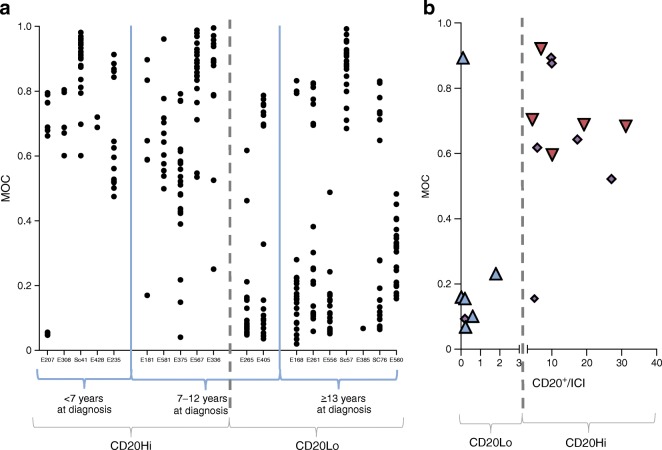
(**a**) Dot plots showing the distribution of the mean MOC for islets within each individual. Individuals were grouped by age at diagnosis (with each age range separated by blue lines) and according to their immune cell profiles (whether CD2Hi or CD20Lo; using the grey dashed line). (**b**) Scatterplot of the number of CD20^+^ cells per ICI arranged by median, and plotted against the mean MOC for islets in each case. Different age ranges indicated by the fill colour: red, <7 years; purple, 7–12 years; blue, ≥13 years

In practice, among those diagnosed at ≥13 years the pattern of proinsulin distribution was more complex in that two distinct subpopulations of residual ICIs were usually present (Figs 2 and 3). As explained above, proinsulin and insulin were confined to separate intracellular compartments in the majority of islets while co-localisation of the antigens was seen in a small number of islets in each case. Thus, with only one exception (Sc57) the presence of these two clearly separable subpopulations of islets was characteristic of individuals diagnosed at the older ages in the pancreas sections studied (Fig. 3).

### Individuals in the intermediate age group at diagnosis (7–12 years) have one or other of the histological phenotypes seen in those diagnosed <7 or ≥13 years

A question arising from these observations is whether the correlation between immune phenotype and proinsulin distribution seen in children diagnosed at <7 years vs those diagnosed ≥13 years was retained within the intermediate age group (i.e. among those diagnosed at 7–12 years). We found that the proinsulin distribution in the ICIs of children aged 7–12 years at diagnosis with a CD20Hi immune profile replicated those diagnosed at <7 years (Figs 2 and 3). By contrast, the pattern seen in children who had a CD20Lo immune profile replicated those diagnosed at ≥13 years (Figs 2 and 3). This segregation of the ‘CD20’ and proinsulin–insulin co-localisation phenotypes (Fig. 3b) supports the conclusion that childhood-onset type 1 diabetes comprises distinct endotypes.

### Proinsulin–insulin co-localisation phenotypes in pancreases from individuals with long-term type 1 diabetes diagnosed at <7 or ≥13 years

Immunohistological studies in longer-term type 1 diabetes (>5 years’ duration) were also undertaken but were inevitably limited by the fact that many fewer residual ICIs are present at the time of death than at disease onset [6]. This is particularly true among those diagnosed at <7 years, where only 17% of individuals retain any ICIs (vs 48% in those diagnosed at ≥13 years; ESM Fig. 1). Therefore, for studies in longer duration patients we used pancreas samples from both the EADB and nPOD collections (ESM Tables 3 and 5).

**Fig. 4:**
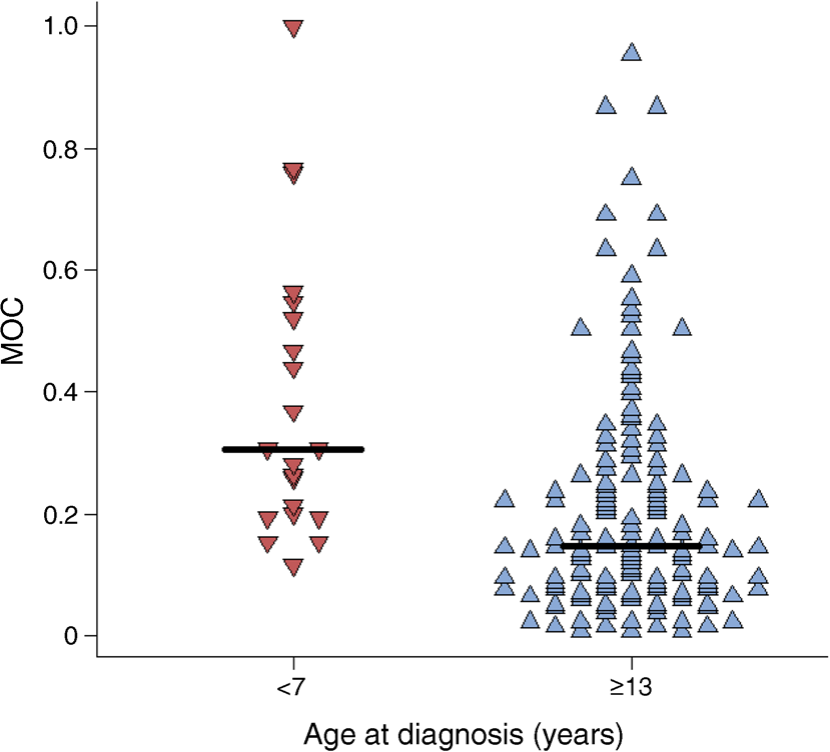
Dot plot showing distribution of the mean MOC for islets in pancreas samples from individuals with long duration of type 1 diabetes (≥5 years’ duration) and originally diagnosed at <7 years (*n* = 3; red) and ≥13 years (*n* = 10; blue). Black bars represent median values for each group

The pattern of proinsulin–insulin co-localisation found in those with longer duration of disease varied from that seen close to diagnosis, with a reduction in the proportion of islets having high proinsulin–insulin co-localisation (Fig. 4). In those diagnosed at ≥13 years, a higher proportion of ICIs persisted (ESM Fig. 1) and proinsulin–insulin co-localisation was minimal (Fig. 4).

### Differences in serum C-peptide and proinsulin in individuals with long-term type 1 diabetes diagnosed at <7 or ≥13 years

**Fig. 5:**
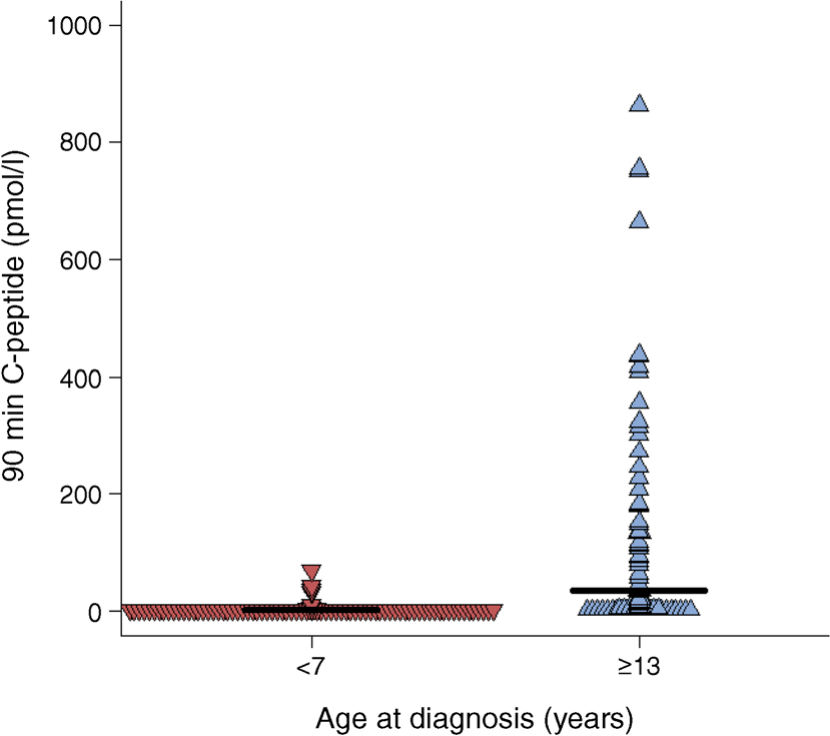
Dot plot showing 90 min stimulated C-peptide values in individuals diagnosed at <7 years (*n* = 87; red) or ≥13 years (*n* = 84; blue). Black bars represent median values for each group

**Fig. 6:**
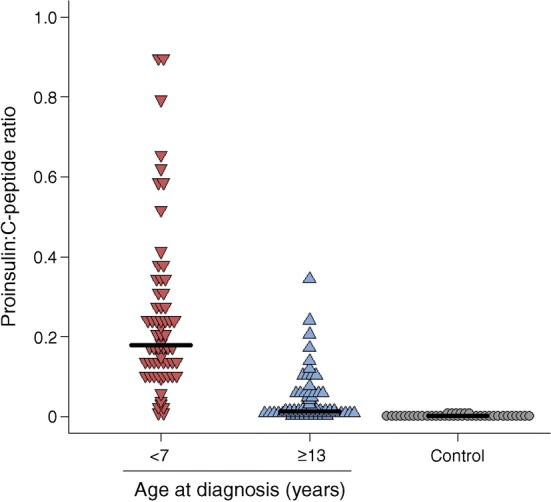
Dot plot showing the proinsulin:C-peptide ratio in individuals with at least one detectable value for those diagnosed with type 1 diabetes <7 years (*n* = 62; red) or ≥13 years (*n* = 70; blue) and for control individuals (*n* = 39; grey). Black bars represent median values for each group

In order to discover whether the histological differences in the pancreas correlate with relevant clinical variables, circulating proinsulin and C-peptide concentrations were measured in individuals with longer-term type 1 diabetes (≥5 years) within the two distinct age groups (diagnosed at <7 years or ≥13 years of age; ESM Table 7). The patients who were diagnosed at <7 years had much lower stimulated C-peptide levels than patients diagnosed at ≥13 years (Fig. 5; median <3 pmol/l, IQR <3 to <3 vs 34.5 pmol/l, IQR <3–151; *p* < 0.0001). Approximately 70% had detectable proinsulin (72% of the <7 years group and 67% of the ≥13 years group, *p* = 0.4) with a median proinsulin level of 0.45 pmol/l (IQR <0.3–0.8) in the <7 years group vs 0.5 pmol/l (IQR <0.3–1.6) (*p* = 0.3) in the >13 years group (ESM Table 7). 62% had detectable proinsulin even in the absence of detectable C-peptide. Strikingly, the median proinsulin:C-peptide ratio was significantly increased in those diagnosed at <7 years compared with individuals diagnosed at ≥13 years (Fig. 6) (0.18, IQR 0.10–0.31 vs 0.01, IQR 0.01–0.10; *p* < 0.0001) and control individuals without diabetes (0.003, IQR 0.002–0.005; *p* < 0.0001). Absolute proinsulin values are shown in ESM Fig. 2. These data suggest that clear phenotypic differences can be detected in the blood of the two type 1 diabetes groups defined by age of diagnosis.

## Discussion

Our results strongly suggest that type 1 diabetes exists as distinct conditions that segregate according to age at diagnosis and are distinguishable histologically. They can be defined by the profile of infiltrating immune cells in inflamed islets and by the extent to which proinsulin and insulin are co-localised within beta cells. We propose that these may represent disease endotypes and suggest that they are defined as type 1 diabetes endotype 1 (T1DE1) and type 1 diabetes endotype 2 (T1DE2). In advancing this proposal, we do not intend to imply that a simple dichotomy will ultimately be sufficient to account for the entire heterogeneity seen in people developing type 1 diabetes. Rather, it is probable that additional endotypes will be defined as further variables are considered. Nevertheless, the present results suggest that future therapeutic trials in type 1 diabetes should be designed to take account of the important aetiopathological differences that are now being revealed. This might be achieved most readily by noting the age at diagnosis. Specifically, we propose that children diagnosed in the earliest years of life may require different immunotherapeutic options vs those who are older at onset.

To verify the existence of endotypes of type 1 diabetes, we focused on the subcellular distribution of proinsulin and mature insulin in the residual beta cells present in individuals diagnosed with type 1 diabetes at different ages and related these to previously described immune phenotypes [8]. This revealed that, among children developing the condition before the age of 7 years (CD20Hi phenotype; T1DE1) there is disruption of insulin processing. In almost all islets (and within all residual beta cells in those islets), significant quantities of the newly synthesised prohormone become co-localised with mature insulin. This situation contrasts markedly with that found in control individuals of equivalent age and in young people developing diabetes after the age of 12 years (T1DE2), where proinsulin was preferentially retained within a perinuclear compartment in most islets and was not co-localised with mature insulin. Thus, we have been able to define disease endotypes that segregate according to age at diagnosis and can be differentiated histologically by alterations in proinsulin processing and immune phenotype.

### Measurements of serum proinsulin reflect the histological appearance

Importantly, we have also been able to correlate new clinical data with the histological findings (albeit in different individuals) to verify our conclusions. Notably, we found that although absolute stimulated C-peptide levels were reduced in the youngest individuals (diagnosed at age <7 years), the ratio of serum proinsulin:C-peptide was elevated in these individuals since proinsulin levels in the circulation were maintained (ESM Fig. 2). This was true even when the individuals were studied more than 5 years after diagnosis. As such, these data are fully consistent with reports that circulating proinsulin levels are highest in children diagnosed below the age of 10 years who are studied at, or soon after, diagnosis [10, 11]. We also show that similarly aged control individuals have a much lower proinsulin:C-peptide ratio (Fig. 6), demonstrating that this is not an effect of age alone. Together, these data imply that, when considered alongside age at diagnosis, measurement of the ratio of proinsulin to C-peptide may represent a convenient biomarker to distinguish the endotypes defined here.

Our data fit with the emerging literature that detectable proinsulin, even in the absence of detectable C-peptide, can occur in type 1 diabetes of long duration [16, 17]. Steenkamp et al [17] described 16% of patients with detectable proinsulin compared with Sims et al [16] who reported 90% with detectable proinsulin. Our data fit most closely with the results of Sims and colleagues [16]; however, differences in the analytical sensitivity of assays and in the cohorts may explain the difference between the studies [16, 17]. To aid comparison, and to inform future clinical studies of proinsulin, we have included the detailed method validation we undertook prior to using the assay in this study (see ESM Validation Method). Our data are also consistent with other evidence implying that the prohormone convertases and carboxypeptidase E involved in proinsulin cleavage may become downregulated in type 1 diabetes [18, 19]. The consequence of such changes is that, rather than being processed fully to yield mature insulin on emergence from the Golgi apparatus in nascent secretory granules, significant amounts of proinsulin persist. This accumulates with insulin and is released into the circulation upon granule exocytosis, leading to a measurable rise in the circulating proinsulin:C-peptide ratio.

### The patterns of proinsulin processing seen in children diagnosed in the intermediate age group (7–12 years) support the existence of disease endotypes

The present conclusion that endotypes of type 1 diabetes exist has arisen from studies of individuals diagnosed with type 1 diabetes at the two extremes of our chosen age ranges. Therefore, it was important to also examine the pancreases of children newly diagnosed with type 1 diabetes between the ages of 7–12 years. These provided further important evidence for independent pathological processes since quantification of the extent of proinsulin–insulin co-localisation allowed this group to be divided into two separate populations. One of these mirrored the features found in the <7 years group while the other had a profile equivalent to that found in those in the ≥13 years group. Importantly, despite being analysed in a blinded manner, all individuals segregated with their previously designated immune cell phenotypes (whether CD20Hi or CD20Lo; Figs 2 and 3). Thus, we were able to establish that the pathological features seen in the pancreases of children diagnosed within the mid-range of ages (between 7–12 years) did not form a continuum but, rather, they segregated with the proposed endotypes.

These observations suggest that the aetiopathology of type 1 diabetes occurs by different pathways in very young children compared with those who are older at diagnosis. The first of these (T1DE1) involves a highly aggressive, hyperimmune attack in which most islets are inflamed and aberrant proinsulin processing occurs. This leads to the co-secretion of both unprocessed and mature insulin, such that the circulating ratio of proinsulin:C-peptide is unusually high. By contrast, a different endotype (T1DE2) is characterised by a reduced intensity of islet autoimmunity, the persistence of higher proportions of ICIs (each with greater numbers of beta cells) and a lower circulating proinsulin:C-peptide ratio.

Very recent data imply that children diagnosed with type 1 diabetes at <7 years or ≥13 years may display differences in genetic predisposition [20, 21]; a finding that is fully consistent with our endotype hypothesis. It cannot be excluded, however, that the immune cell and proinsulin processing profiles could be influenced by other physiological changes occurring during development (e.g. the onset of puberty) rather than solely by a differing genetic architecture. In either case, it is abundantly clear that very different outcomes derive from the autoimmune process in children diagnosed with type 1 diabetes <7 years vs those >13 years.

### Individuals with T1DE2 have two populations of islets

Clinical studies have shown that a significant proportion of people with type 1 diabetes continue to secrete insulin for many years beyond diagnosis [22–24], although this is rare in children diagnosed <7 years (Fig. 5). In support of these findings, we have recently discovered that the rate of C-peptide decline seen early in the disease course is markedly attenuated, and effectively ceases, from about 7 years after onset [25]. Thus, even people with very long-standing disease may retain ICIs which secrete small amounts of insulin [6, 26, 27]. In view of this, we explored whether the aberrant pattern of proinsulin:insulin co-localisation seen at diagnosis is maintained in residual ICIs over time. Recent data from others [19], as well as our current histological results, support this. We also noted that the proportion of islets in which proinsulin and insulin were co-localised was reduced in individuals with longer duration of disease, even among those who had been diagnosed initially before the age of 7 years (Fig. 4). This implies that the population of ICIs that persists in the longer term mainly comprises those islets that are least prone to the abnormalities of proinsulin processing reported here, and are spared preferentially during the autoimmune attack. Alternatively, it is also possible that individuals with more complete proinsulin processing at baseline have improved survival of beta cells overall, leading to higher numbers of residual insulin positive islets over time. Further studies will be required to resolve these possibilities.

### Limitations

We are aware that the current study has certain limitations; not least that the number of samples examined for proinsulin processing in each age group is small. However, we would emphasise that such studies are limited by the availability of suitably fixed and processed pancreas samples from people with recent-onset disease. Indeed, using a cut-off of 2 years post diagnosis and childhood onset of disease, very few suitable samples are available worldwide [6] and we have studied the majority of these; which is a clear strength. Moreover, the differences in the immune cell and proinsulin processing profiles between the age groups is stark and provides compelling support for the existence of disease endotypes.

A second potential weakness is that many of the individuals with type 1 diabetes died in ketoacidosis (ESM Table 1), meaning that their islets were subject to metabolic stress. However, the fact that all individuals within the intermediate age range (7–12 years) died in diabetic ketoacidosis but their proinsulin profiles segregated precisely with their islet immune phenotype (Fig. 2) implies that these differences were unlikely to have been caused directly by the presence of diabetic ketoacidosis.

Third, we have not studied proinsulin processing in the pancreases of older adults at the onset of type 1 diabetes [28]. We cannot, therefore, extrapolate beyond the childhood disease. This is important since a recent histopathological study has reported differences in islet cell proinsulin distribution in correlation with islet cell autoantibody status in adults who had not progressed to diabetes [29] and heterogeneity in adult-onset autoimmune diabetes has been reported [30]. Despite this, and in accord with our observations in people diagnosed in their teenage years, proinsulin was still clearly distinguishable within the perinuclear region of an islet of an adult with newly diagnosed type 1 diabetes who had undergone pancreatic biopsy [29]. We should also note that, although we have occasionally seen individual beta cells in control islets that immunostain very strongly for proinsulin in the cytoplasm, the frequency at which these appear is much lower than that seen in the islets of children with type 1 diabetes (Fig. 2).

### Implications of the study

Importantly, age-dependent differences in both rates of C-peptide decline and drug responses have been seen in several clinical trials in type 1 diabetes [2, 31–37] and this highlights the possibility of stratification according to age at diagnosis. Disease endotypes have also been inferred in separate studies of the temporal sequence of islet autoantibody seroconversion in The Environmental Determinants of Diabetes in the Young (TEDDY) study. In particular, a recent cluster analysis of TEDDY data implies that children who develop combinations of autoantibodies to insulin and islet antigen 2 (IA2) (with or without GAD65) within the first 2 years of life are likely to progress to diabetes within 5 years (i.e. before the age of 7 years) [38]. Thus, it seems likely that these may correspond to children with the T1DE1 endotype defined here. Future therapeutic strategies will need to take account of such differences if they are to be effective.

### Electronic supplementary material

ESM 1 (PDF 565 kb)

## Data Availability

The datasets generated during and/or analysed during the current study are available from the corresponding authors on reasonable request.

## Appendix

Members of the TIGI study team: Bart O. Roep (Leiden University Medical Center, Leiden, Netherlands & Department of Diabetes Immunology, Diabetes & Metabolism Research Institute, Beckman Research Institute, City of Hope, CA, USA); Timothy I. Tree (Department of Immunobiology, Faculty of Life Sciences and Medicine, King’s College London, London, UK); Kashyap Patel, Suzy Hammersley, Robert Bolt and Anita V. Hill (all NIHR CRF, University of Exeter Medical School, Exeter, UK).

## Abbreviations

EADB: Exeter Archival Diabetes Biobank
ICI: Insulin-containing islet
MOC: Manders overlap coefficient
nPOD: Network of Pancreatic Organ Donors
T1DE1: Type 1 diabetes endotype 1
T1DE2: Type 1 diabetes endotype 2
TIGI: Type 1 diabetes, Immunology, Genetics and endogenous Insulin production
TEDDY: The Environmental Determinants of Diabetes in the Young

## References

[1] Skyler JS, Bakris GL, Bonifacio E (2017). Differentiation of diabetes by pathophysiology, natural history, and prognosis. Diabetes.

[2] DiMeglio LA, Evans-Molina C, Oram RA (2018). Type 1 diabetes. Lancet.

[3] Atkinson M, Roep BO, Posgai A, Wheeler DCS, Peakman M (2018). The challenge of modulating β-cell autoimmunity in type 1 diabetes. Lancet Diabetes Endocrinol.

[4] Herold KC, Bundy BN, Long SA (2019). An anti-CD3 antibody, teplizumab, in relatives at risk for type 1 diabetes. N Engl J Med.

[5] Foulis AK, Liddle CN, Farquharson MA, Richmond JA, Weir RS (1986). The histopathology of the pancreas in type 1 (insulin-dependent) diabetes mellitus: a 25-year review of deaths in patients under 20 years of age in the United Kingdom. Diabetologia.

[6] Morgan NG, Richardson SJ (2018) Fifty years of pancreatic islet pathology in human type 1 diabetes: insights gained and progress made. Diabetologia 61:2499–2506 10.1007/s00125-018-4731-y PMC6223849 30255378

[7] Battaglia M, Ahmed S, Anderson MS (2020). Introducing the endotype concept to address the challenge of disease heterogeneity in type 1 diabetes. Diabetes Care.

[8] Leete P, Willcox A, Krogvold L (2016). Differential insulitic profiles determine the extent of beta cell destruction and the age at onset of type 1 diabetes. Diabetes.

[9] Arif S, Leete P, Nguyen V (2014). Blood and islet phenotypes indicate immunological heterogeneity in type 1 diabetes. Diabetes.

[10] Sims EK, Chaudhry Z, Watkins R (2016). Elevations in the fasting serum proinsulin-to-C-peptide ratio precede the onset of type 1 diabetes. Diabetes Care.

[11] Watkins RA, Evans-Molina C, Terrell JK (2016). Proinsulin and heat shock protein 90 as biomarkers of beta-cell stress in the early period after onset of type 1 diabetes. Transl Res.

[12] Roder ME, Knip M, Hartling SG, Karjalainen J, Akerblom HK, Binder C (1994). Disproportionately elevated proinsulin levels precede the onset of insulin-dependent diabetes mellitus in siblings with low first phase insulin responses. The Childhood Diabetes in Finland Study Group. J Clin Endocrinol Metab.

[13] Willcox A, Richardson SJ, Bone AJ, Foulis AK, Morgan NG (2009). Analysis of islet inflammation in human type 1 diabetes. Clin Exp Immunol.

[14] Bolte S, Cordelières FP (2006). A guided tour into subcellular colocalization analysis in light microscopy. J Microsc.

[15] Manders EMM, Verbeek FJ, Aten JA (1993). Measurement of co-localization of objects in dual-colour confocal images. J Microsc.

[16] Sims EK, Bahnson HT, Nyalwidhe J (2019). Proinsulin secretion is a persistent feature of type 1 diabetes. Diabetes Care.

[17] Steenkamp DW, Cacicedo JM, Sahin-Efe A, Sullivan C, Sternthal E (2017). Preserved proinsulin secretion in long-standing type 1 diabetes. Endocr Pract.

[18] Wasserfall C, Nick HS, Campbell-Thompson M (2017). Persistence of pancreatic insulin mRNA expression and proinsulin protein in type 1 diabetes pancreata. Cell Metab.

[19] Sims EK, Syed F, Nyalwidhe J (2019). Abnormalities in proinsulin processing in islets from individuals with longstanding T1D. Transl Res.

[20] Inshaw JRJ, Cutler AJ, Crouch DJM, Wicker LS, Todd JA (2019). Genetic variants predisposing most strongly to type 1 diabetes diagnosed under age 7 years lie near candidate genes that function in the immune system and in pancreatic β-cells. Diabetes Care.

[21] Redondo MJ, Concannon P (2020). Genetics of type 1 diabetes comes of age. Diabetes Care.

[22] Oram RA, Jones AG, Besser RE (2014). The majority of patients with long-duration type 1 diabetes are insulin microsecretors and have functioning beta cells. Diabetologia.

[23] Davis AK, DuBose SN, Haller MJ (2015). Prevalence of detectable C-peptide according to age at diagnosis and duration of type 1 diabetes. Diabetes Care.

[24] Wang L, Lovejoy NF, Faustman DL (2012). Persistence of prolonged C-peptide production in type 1 diabetes as measured with an ultrasensitive C-peptide assay. Diabetes Care.

[25] Shields BM, McDonald TJ, Oram R (2018). C-peptide decline in type 1 diabetes has two phases: an initial exponential fall and a subsequent stable phase. Diabetes Care.

[26] Keenan HA, Sun JK, Levine J (2010). Residual insulin production and pancreatic beta-cell turnover after 50 years of diabetes: Joslin Medalist Study. Diabetes.

[27] Battaglia M, Atkinson MA (2015). The streetlight effect in type 1 diabetes. Diabetes.

[28] Thomas NJ, Jones SE, Weedon MN, Shields BM, Oram RA, Hattersley AT (2018). Frequency and phenotype of type 1 diabetes in the first six decades of life: a cross-sectional, genetically stratified survival analysis from UK Biobank. Lancet Diabetes Endocrinol.

[29] Rodriguez-Calvo T, Zapardiel-Gonzalo J, Amirian N (2017). Increase in pancreatic proinsulin and preservation of beta-cell mass in autoantibody-positive donors prior to type 1 diabetes onset. Diabetes.

[30] Buzzetti R, Zampetti S, Maddaloni E (2017). Adult-onset autoimmune diabetes: current knowledge and implications for management. Nat Rev Endocrinol.

[31] Wherrett DK, Chiang JL, Delamater AM (2015). Defining pathways for development of disease-modifying therapies in children with type 1 diabetes: a consensus report. Diabetes Care.

[32] Keymeulen B, Walter M, Mathieu C (2010). Four-year metabolic outcome of a randomised controlled CD3-antibody trial in recent-onset type 1 diabetic patients depends on their age and baseline residual beta cell mass. Diabetologia.

[33] Hagopian W, Ferry RJ, Sherry N (2013). Teplizumab preserves C-peptide in recent-onset type 1 diabetes: two-year results from the randomized, placebo-controlled Protege trial. Diabetes.

[34] Rigby MR, DiMeglio LA, Rendell MS (2013). Targeting of memory T cells with alefacept in new-onset type 1 diabetes (T1DAL study): 12 month results of a randomised, double-blind, placebo-controlled phase 2 trial. Lancet Diabetes Endocrinol.

[35] Rigby MR, Harris KM, Pinckney A (2015). Alefacept provides sustained clinical and immunological effects in new-onset type 1 diabetes patients. J Clin Invest.

[36] Orban T, Bundy B, Becker DJ (2011). Co-stimulation modulation with abatacept in patients with recent-onset type 1 diabetes: a randomised, double-blind, placebo-controlled trial. Lancet.

[37] Pescovitz MD, Greenbaum CJ, Krause-Steinrauf H (2009). Rituximab, B-lymphocyte depletion, and preservation of beta-cell function. N Engl J Med.

[38] Krischer JP, Lynch KF, Lernmark A (2017). Genetic and environmental interactions modify the risk of diabetes-related autoimmunity by 6 years of age: the TEDDY Study. Diabetes Care.

