# Supplementary material for: Studies of insulin and proinsulin in pancreas and serum support the existence of aetiopathological endotypes of type 1 diabetes associated with age at diagnosis
Source: Diabetologia. 2020 Mar 15;63(6):1258–67. doi: 10.1007/s00125-020-05115-6 (PMC7228905; doi:10.1007/s00125-020-05115-6)
Supplement: Supplementary file 1 — (PDF 565 kb) [file 125_2020_5115_MOESM1_ESM.pdf]

ESM Figure 1

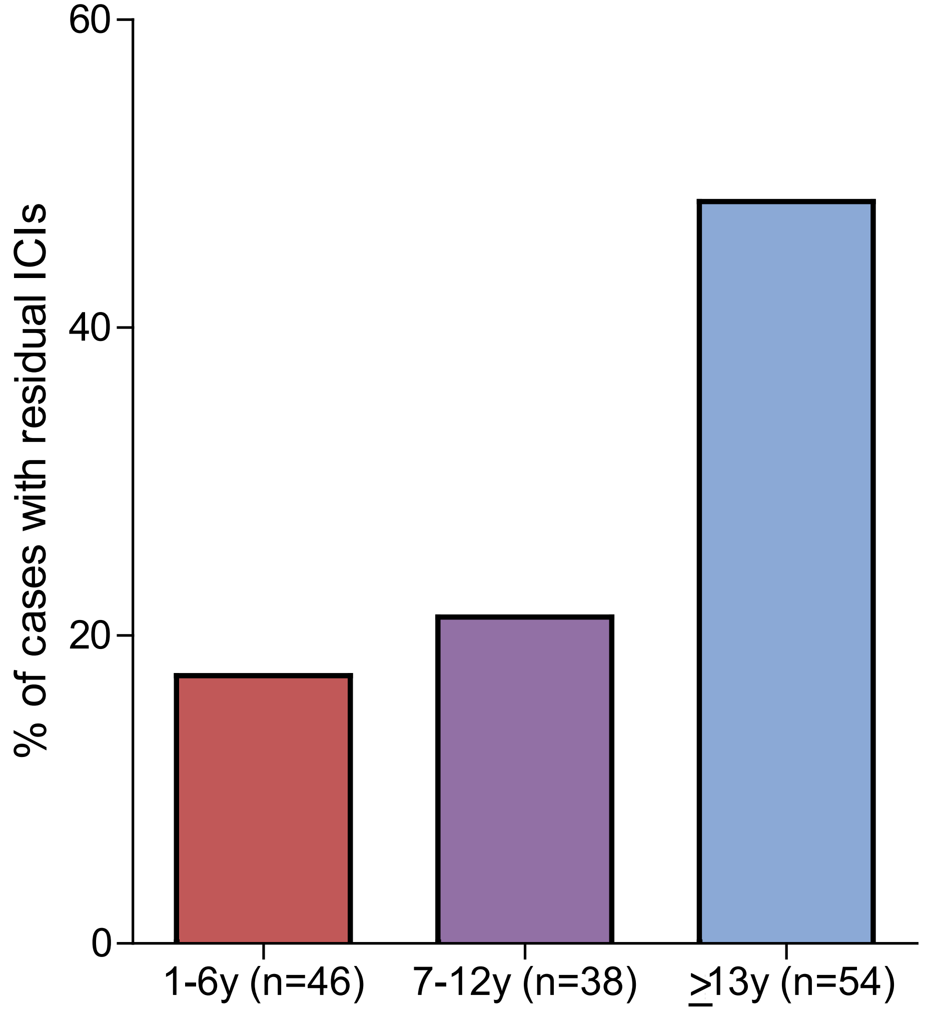

Proportion of patients who retain insulin positive islets at ≥5y duration of disease subdivided according to their age at diagnosis in years.

ESM Figure 2

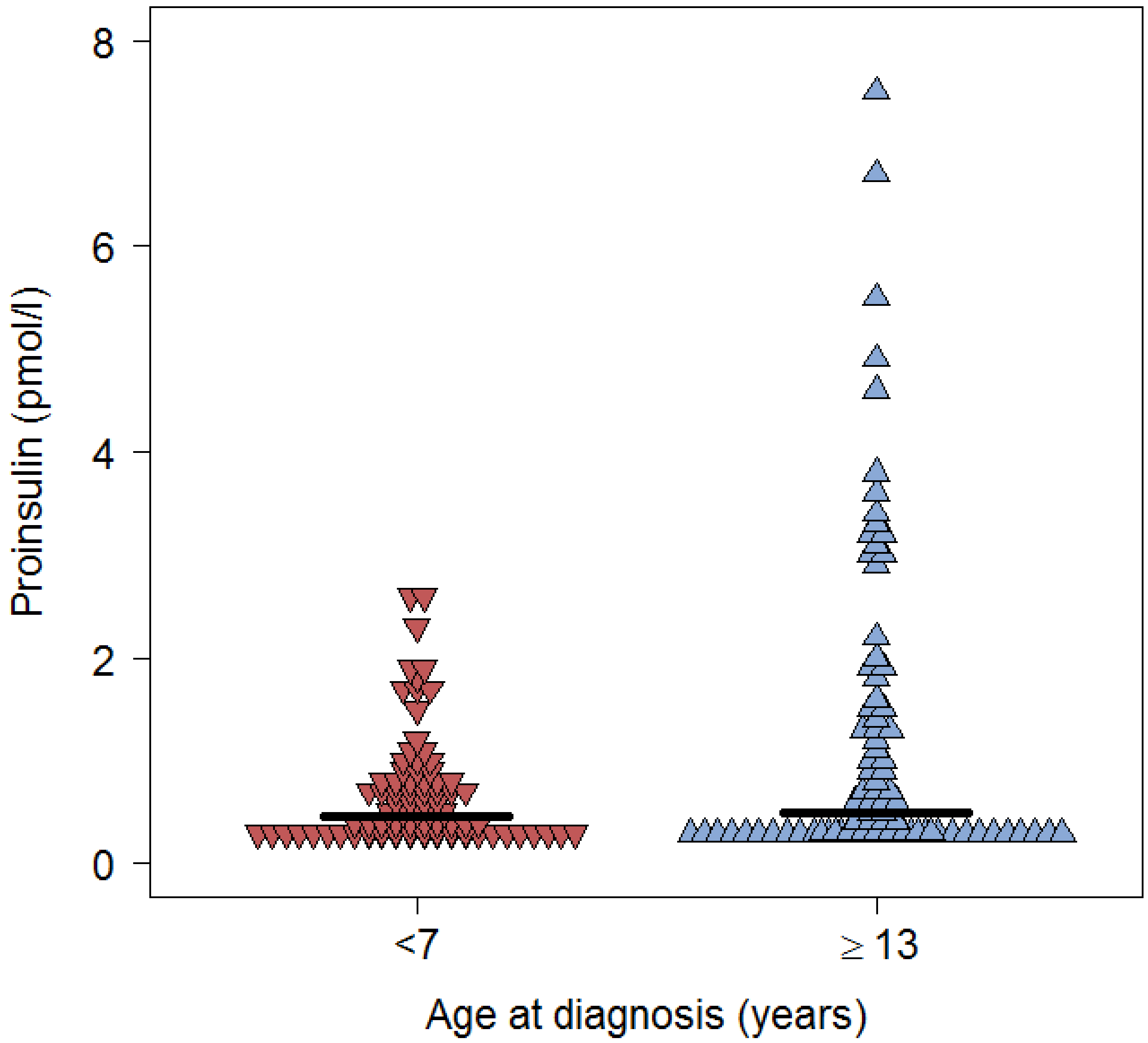

Absolute values for circulating proinsulin in subjects diagnosed with type 1 diabetes at increasing ages.
Median values are indicated with black lines

Supplementary Table 1: Donor information for T1D cases where immunophenotype was characterised.

Case ID	Biobank	Age at Diagnosis (y)	Duration of disease (y)	Gender	Cause of death
E308	EADB	3	0.02	F	DKA
E207	EADB	3	0.06	F	N/A
SC41	EADB	4	0.06	F	DKA
E428	EADB	5	0.02	M	DKA
E235	EADB	6	0.27	M	DKA
E181	EADB	7	0.01	F	DKA
E265	EADB	7	0.14	F	DKA
E405	EADB	8	0.01	F	DKA
E581	EADB	10	0.02	F	DKA
E375	EADB	11	0.01	F	DKA
E567	EADB	11	0	F	DKA
E336	EADB	12	0.16	F	DKA
E261	EADB	18	0.06	F	DKA
SC57	EADB	18	0.02	F	DKA
E168	EADB	18	0.16	M	Hypoglycaemia
E556	EADB	18	0.33	M	Hypoglycaemia
E385	EADB	19	1.5	F	DKA and pneumonia
SC76	EADB	20	0.06	M	DKA
E560	EADB	42	1.5	F	Glioma

N/A – not available; DKA – Diabetic ketoacidosis

Supplementary Table 2: Donor information for Control cases used for the recent onset (<2y) proinsulin study

Case ID	Biobank	Age at Diagnosis (y)	Gender	Cause of death
240/90	EADB	2	M	Haemophilus influenza meningitis
242/89	EADB	3	M	Meningococcal septicaemia
184/90	EADB	5	M	Unknown
245/90	EADB	6	M	Pneumonia
315/89	EADB	9	M	Intussusception
333/66	EADB	16	M	Thymomae haemorrhage
191/67	EADB	25	M	Post-op death (colectomy)
65/71	EADB	40	M	Stroke (CVA)

Supplementary Table 3: Donor information for T1D cases used for the long duration (>5y) proinsulin study

Case ID	Biobank	Age at diagnosis (y)	Duration of disease (y)	Gender	Cause of death
6049	nPOD	5	10.0	F	Anoxia
10382	EADB	5	6.0	M	DKA
6302	nPOD	6	32.5	M	Anoxia
E558	EADB	13	12.0	F	N/A
6306	nPOD	14	5.0	M	Head trauma
6195	nPOD	14.2	5.0	M	Head trauma
6070	nPOD	15	7.0	F	Anoxia
6081	nPOD	16.4	15.0	M	Cerebrovascular/ stroke
SC95	EADB	17	5.0	F	DKA
6038	nPOD	17.2	20.0	F	Anoxia
6328	nPOD	19	20.0	M	Anoxia
6040	nPOD	30	20.0	F	Cerebrovascular/ stroke
6307	nPOD	35	10.0	F	Anoxia

Supplementary Table 4. Available clinical characteristics of patients in the EADB recent onset cohort (<2y since diagnosis) analysed for proinsulin:insulin localisation. Data presented as n (%) or median (inter-quartile range)

Disease duration <2y (grouped by age at diagnosis)			
	**<7y (n=5)**	**7-12y (n=7)**	**≥13y (n=6)**
Male	2 (40%)	0	3 (50%)
Age at diagnosis (y)	4 (3, 5)	10 (7.5, 11)	18 (18, 19.5)
Duration of diabetes (y)	0.06 (0.06, 0.08)	0.02 (0.01, 0.06)	0.06 (0.02, 0.17)

Supplementary Table 5. Available clinical characteristics of patients in the EADB longer duration cohort (>5y since diagnosis). Data presented as n (%) or median (inter-quartile range)

Disease duration longer >5y (grouped by age at diagnosis)		
	**<7y (n=3)**	**≥13y (n=10)**
Male	3 (100%)	4 (44%)
Age at diagnosis (y)	5 (5, 5.5)	16.5 (14.25, 18.5)
Age at study (y)	15.0 (13, 26.75)	28 (22, 38.5)
Duration of diabetes (y)	10 (8, 21.25)	11 (5.5, 18.75)

Supplementary table 6. Antibody details.

Antibody	Company	Cat No	Clone	Species	[Stock]	Dilution	Incubation time
Insulin	Dako	A0564	Polyclonal	Guinea-Pig	14.4mg/ml	1/343 (HRP and IF)	1h (RT)
Proinsulin	Abcam	ab8301	3A1	Mouse	2mg/ml	1/500 (IF)	2h (RT)
CD20cy	Dako	M0755	L26	Mouse	0.2mg/ml	1/1500 (HRP); 1/800 (TSA IF)	1h (RT) ; Overnight (4C)
CD4	Abcam	ab846	BC/1F6	Mouse	25mg/ml	1/25 (HRP)	Overnight 4C
CD4	Abcam	ab133616	EPR6855	Rabbit	0.054mg/ml	1/100 (TSA IF)	2 (RT)
CD8	Abcam	ab4055	Polyclonal	Rabbit	0.2mg/ml	1/400 (HRP)	Overnight 4C
CD8	Dako	M7103	(C8/144B)	Mouse	0.157mg/ml	1/70 (2h or ON TSA IF)	2h or TSA

Supplementary Table 7. Clinical characteristics of patients in the TIGI cohort. Data presented as n (%) or median (inter-quartile range).

	**Age at diagnosis subgroup**	
	**<7y (n=87)**	**≥13y (n=84)**
Male	54 (62%)	37 (44%)
Age at diagnosis (y)	3.1 (2.1, 4.9)	18.5 (14.8, 22.0)
Age at study (y)	16.0 (12.2, 22.3)	35.5 (28.8, 46.2)
Duration of diabetes (y)	12.4 (8.8, 18.6)	15.8 (9.6, 27.6)
BMI age specific z score	1.1 (0.02, 1.6)	0.75 (0.23, 1.41)
HbA1c (mmol/mol)	67 (58, 76)	67 (58, 78)
HbA1c (%)	8.3 (7.5, 9.1)	8.3 (7.5, 9.3)
Insulin Dose (u/kg/day)	0.8 (0.7, 1.0)	0.7 (0.5, 0.9)
C-peptide pmol/l	<3 (<3, <3)	34.5 (<3, 151)
Detectable C-peptide n (%)	13 (15%)	62 (74%)
Proinsulin pmol/l	0.45 (<0.3, 0.8)	0.5 (<0.3, 1.6)
Detectable proinsulin n (%)	63 (72%)	56 (67%)

Proinsulin Assay Workup and Validation

AIM

To assess the performance characteristics of the TECO Medical Intact Proinsulin ELISA kit in use on the Dynex DS2 analyser, by use of various precision studies and recovery experiments.

VALIDATION PLAN

The manufacturer provided a thorough assessment of the assay performance so the plan aimed to replicate these tests, as far as practicable. Alongside these tests some further performance characteristics were determined, such as the Limit of Quantification, the effect of repeated Freeze/Thaw cycles and possible interference from Haemolysis. The studies were performed by analysis of a combination of plasma, serum, supplied calibration material and International Standard reference material. The results are given below. It is recommended that each ELISA plate is read at 2 wavelengths, 405nm and 450nm, the latter showing greater sensitivity at the lower range, so each test has been performed and reported from readings in both wavelengths for comparison.

Quality Control

Quality Control material provided with the supplied kit was analysed in each assay run to assess the validity of the assay run. The concentrations of proinsulin obtained were compared with those of the supplied data.

CONTROL 1					
Plate 2		Plate 3			
405nm	450nm	405nm	450nm	Target Mean	Acceptable Range
11.635	11.474	11.221	11.208	10.6	6.9-14.3
11.976	11.830	11.417	11.419		

Table 1. Proinsulin concentration obtained of Control 1 (pmol/L).

CONTROL 2					
Plate 2		Plate 3			
405nm	450nm	405nm	450nm	Target Mean	Acceptable Range
21.811	21.766	21.982	21.986	21.6	16.2-27.0
21.702	21.615	22.269	22.234		

Table 2. Proinsulin concentration obtained of Control 2 (pmol/L).

All Quality control was within the acceptable ranges of the manufacturers.

Interassay precision

Due to only 2 plates being run in this validation, there is not sufficient data to calculate the Interassay performance. This is a performance characteristic that will require revisiting once further plates have been run, for example examining the values of the quality control material analysed.

Intraassay precision

Human Proinsulin WHO International Standard (NIBSC code 83/500) was reconstituted made up using a protein matrix solution (MultiAssay Diluent, Roche) in order to replicate human plasma. The International Standard was made up to three levels across the given performance range. Analysis of each homogenous mix was repeated 10 times. Given these values precision statistics could then be calculated.

N=10	Mean value (pmol/L)	Standard Deviation	CV (%)
Sample 1 (approx. 5 pmol/L)	4.910	0.369	7.51
Sample 2 (approx. 25 pmol/L)	21.450	0.584	2.72
Sample 3 (approx. 100 pmol/L)	85.275	3.102	3.64

Table 3. Intraassay Precision statistics, read at 405 nm.

N=10	Mean value (pmol/L)	Standard Deviation	CV (%)
Sample 1 (approx. 5 pmol/L)	4.725	0.173	3.66
Sample 2 (approx. 25 pmol/L)	21.424	0.560	2.62

Table 4. Intraassay Precision statistics, read at 450nm. Only 2 samples are given for 450nm read as sample 3 is greater than the measuring range at this wavelength.

From this study it can be seen that at all 3 tested levels all of the Coefficient of Variations (CV) are within the generally accepted limit of 10%. All levels, except Sample 1 at 405nm, perform well with CVs at less than 5%. From these data it can be noted that when the plate is read at 450nm the precision is greater at the lower end of the range than when read at 405nm. The precision of Sample 2 is comparable between the two different read wavelengths.

Limit of Detection

The zero standard provided with the assay kit was analysed with 10 repetitions, to calculate the potential noise, below the detection limit. The quoted limit of detection at plus 2 standard deviations is given as mL.

N=10	Mean OD	Standard Deviation	CV (%)
Calibrator A 0 pmol/L	0.038	0.003	7.28

Table .5 Limit of Detection Data read at 405nm.

N=10	Mean OD	Standard Deviation	CV (%)
Calibrator A 0 pmol/L	0.053	0.005	9.58

Table 6. Limit of Detection Data read at 450nm.

Both of the CVs are less than 10%.

Limit of Quantification

The first standard with a non zero value is run for 10 repetitions and statistics calculated to validate the precision at the lowest end of the assay range, indicating what can be a reliable range for clinical reporting. Standard B, with the given value of 2.5 pmol/L was analysed on the first experimental run.

N=10	Mean OD	Standard Deviation	CV (%)	N=8	Mean concentration (pmol/L)	Standard Deviation	CV (%)
Calibrator B 2.5 pmol/L	0.069	0.004	5.58	Calibrator B 2.5 pmol/L	2.263	0.330	14.58

Table 7. Limit of Quantification, Calibrator B (2.5 pmol/L), both ODs and concentrations given, read at 405nm.

N=10	Mean OD	Standard Deviation	CV (%)	N=8	Mean concentration (pmol/L)	Standard Deviation	CV (%)
Calibrator B 2.5 pmol/L	0.151	0.011	7.35	Calibrator B 2.5 pmol/L	2.597	0.323	12.43

Table 8. Limit of Quantification, Calibrator B (2.5 pmol/L), both ODs and concentrations given, read at 450nm.

At this level, with the plate read at both wavelengths both CVs are greater than the acceptable limits of 10%. The experiment was repeated using calibrator B, but also moving up to the next calibrator C at 8 pmol/L

N=10	Mean OD	Standard Deviation	CV (%)	N=8	Mean concentration (pmol/L)	Standard Deviation	CV (%)
Calibrator B 2.5 pmol/L	0.068	0.009	12.86	Calibrator B 2.5 pmol/L	2.061	0.688	33.36

Table 9. Limit of Quantification, Repeat Calibrator B (2.5 pmol/L), both ODs and concentrations given, read at 405nm.

N=10	Mean OD	Standard Deviation	CV (%)	N=8	Mean concentration (pmol/L)	Standard Deviation	CV (%)
Calibrator B 2.5 pmol/L	0.158	0.023	14.54	Calibrator B 2.5 pmol/L	2.502	0.614	24.52

Table 10. Limit of Quantification, Repeat Calibrator B (2.5 pmol/L), both ODs and concentrations given, read at 450nm.

N=10	Mean OD	Standard Deviation	CV (%)	N=8	Mean concentration (pmol/L)	Standard Deviation	CV (%)
Calibrator C 8 pmol/L	0.150	0.004	2.80	Calibrator C 8 pmol/L	7.846	0.239	3.04

Table 11. Limit of Quantification, Calibrator C (8 pmol/L), both ODs and concentrations given, read at 405nm.

N=10	Mean OD	Standard Deviation	CV (%)	N=8	Mean concentration (pmol/L)	Standard Deviation	CV (%)
Calibrator C 8 pmol/L	0.409	0.008	2.06	Calibrator C 8 pmol/L	8.717	0.173	2.12

Table 12. Limit of Quantification, Calibrator C (8 pmol/L), both ODs and concentrations given, read at 450nm.

From these data it can be seen that the lower Limit of Quantification is unacceptable at the level of Calibrator B and would suggest that to have confidence in a reliable reporting rage the lower limit should be from Calibrator C level, with these specific calibrator values 8 pmol/L.

To extend the reporting range at the lower end it is possible to use the data from the Intraassay Precision test to apply to the Limit of Quantification, specifically at 450nm, where at 5pmol/L the measured CV was 3.66%.

This could be further reinforced in the future by analysis of calibration material made up to a concentration of 5 pmol/L.

When using both wavelengths in combination this would give a measuring range of 5-40pmol/L at 450nm and 8-100pmol/L at 405nm.

Upper limit of Detection

To determine the upper limit of detection and consequently an upper reporting limit the highest calibrator was analysed repeatedly, and precision statistics calculated.

N=10	Mean OD	Standard Deviation	CV (%)	N=8	Mean Proinsulin concentration (pmol/L)	Standard Deviation	CV (%)
Calibrator F 100 pmol/L	1.242	0.023	1.815	Calibrator F 100 pmol/L	101.665	2.311	2.27

Table 13. Upper Limit of Detection, Calibrator F (100 pmol/L), both ODs and concentrations given, read at 405nm

N=10	Mean OD	Standard Deviation	CV (%)	N=8	Mean Proinsulin concentration (pmol/L)	Standard Deviation	CV (%)
Calibrator E 40 pmol/L	1.740	0.054	3.116	Calibrator E 40 pmol/L	38.696	1.054	2.72

Table 14. Limit of Detection, Calibrator E (40 pmol/L), both ODs and concentrations given, read at 450nm

At both wavelengths the CV's are good at 2.27% and 2.72%. This indicates that results can confidently be reported up to 40 pmol/L at 450nm and 100pmol/L at 405nm.

Recovery Test

Plasma and serum patient samples were spiked with known concentration of proinsulin (10pmol/L proinsulin), with the projected concentration calculated and compared to the observed values.

RECOVERY PLASMA

	Neat Conc (pmol/L)	Projected Conc (pmol/L)	Observed Conc (pmol/L)	Recovery (%)
A	12.378	22.378	20.402	91.2
B	12.528	22.528	19.269	85.5
C	9.716	19.716	19.917	101.0
D	31.611	41.611	43.538	104.6

RECOVERY SERUM

	Neat Conc (pmol/L)	Projected Conc (pmol/L)	Observed Conc (pmol/L)	Recovery (%)
A	4.906	14.906	13.953	93.6
B	2.618	12.618	12.626	100.1
C	1.224	11.224	11.872	105.8
D	30.051	40.051	41.904	104.6

Table 15. Projected and observed proinsulin concentrations, with percentage recovery on addition of 10pmol/L proinsulin, in plasma and serum measured at 405nm.

RECOVERY PLASMA

	Neat Conc (pmol/L)	Projected Conc (pmol/L)	Observed Conc (pmol/L)	Recovery (%)
A	12.216	22.216	20.364	91.7
B	12.343	22.343	19.230	86.1
C	9.752	19.752	19.871	100.6
D	31.544	41.544	43.049	103.6

RECOVERY SERUM

	Neat Conc (pmol/L)	Projected Conc (pmol/L)	Observed Conc (pmol/L)	Recovery (%)
A	4.943	14.943	14.123	94.5
B	2.716	12.716	12.759	100.3
C	1.246	11.246	12.058	107.2
D	30.177	40.177	41.635	103.6

Table 16. Projected and observed proinsulin concentrations, with percentage recovery on addition of 10pmol/L proinsulin, in plasma and serum measured at 450nm.

The recovery shown is generally excellent with the majority of samples demonstrating recovered values within +/-10% of the projected value.

Dilution Test

Test patients plasma and serum was diluted to 2 concentrations and the projected proinsulin concentration compared to the observed concentration. The diluent used was protein matrix solution (MultiAssay Diluent, Roche).

DILUTION TEST PLASMA

	Dilution Factor	Expected Conc (pmol/l)	Observed Conc (pmol/L)	Recovery (%)
Plasma A	1	12.38	12.38	100.00
	2	6.19	6.35	102.52
	4	3.09	0.31	10.15
Plasma B	1	12.53	12.53	100.00
	2	6.26	5.85	93.31
	4	3.13	0.86	27.55
Plasma C	1	9.72	9.72	100.00
	2	4.86	5.33	109.63
	4	2.43	0.71	29.07*
Plasma D	1	31.61	31.61	100.00
	2	15.81	20.87	132.07
	4	7.90	6.68	84.53

DILUTION TEST SERUM

	Dilution Factor	Expected Conc (pmol/l)	Observed Conc (pmol/L)	Recovery (%)
Serum A	1	4.91	4.91	100.00
	2	2.45	2.30	93.89*
	4	1.23	0.82	66.53*
Serum B	1	2.62	2.62	100.00
	2	1.31	0.94	71.81*
	4	0.65	0.43	65.70*
Serum C	1	1.22	1.22	100.00*
	2	0.61	0.27	44.77*
	4	0.31	Incalculable	No value*
Serum D	1	30.05	30.05	100.00
	2	15.03	15.91	105.90
	4	7.51	6.98	92.91

Table 17. Projected and observed proinsulin concentrations with varying dilution factors, with percentage recovery shown, both in plasma and serum, read at 405nm.

DILUTION TEST PLASMA

	Dilution Factor	Projected Conc (pmol/l)	Observed Conc (pmol/L)	Recovery (%)
Plasma A	1	12.22	12.22	100.00
	2	6.11	6.73	110.20
	4	3.05	0.55	18.01
Plasma B	1	12.34	12.34	100.00
	2	6.17	6.28	101.82
	4	3.09	1.15	37.20
Plasma C	1	9.75	9.75	100.00
	2	4.88	5.77	118.23
	4	2.44	1.04	42.78*
Plasma D	1	31.54	31.54	100.00
	2	15.77	21.12	133.91
	4	7.89	7.02	88.98

DILUTION TEST SERUM

	Dilution Factor	Projected Conc (pmol/l)	Observed Conc (pmol/L)	Recovery (%)
Serum A	1	5.60	5.60	100.00
	2	2.80	2.76	98.46
	4	1.40	1.22	87.17*
Serum B	1	2.72	2.72	100.00
	2	1.36	1.36	100.29*
	4	0.68	0.83	122.09*
Serum C	1	1.25	1.25	100.00*
	2	0.62	0.70	111.88*
	4	0.31	0.11	34.03*
Serum D	1	30.16	30.18	100.00
	2	15.09	16.25	107.69
	4	7.54	7.39	97.90

Table 18. Projected and observed proinsulin concentrations with varying dilution factors, with percentage recovery shown, both in plasma and serum, read at 450nm.

NB. Results marked with have projected and/or initial concentrations outside the manufacturers designated assay range of 2.5-100pmol/L.*

The dilution test show that in most observations a dilution factor of 2 will generally yield a comparable result. A dilution factor of 4 is, in most of the observed tests, not comparable. Considerations have to be made to the lower limit of detection and the amount of error that may be attributed to this. Also dilutions were made up by hand, which may also carry a portion of error.

Cross reactivity

As the proinsulin molecule is precursor to C-peptide and insulin, cross reactivity with these proteins have been tested to detect any competition or interference. Protein matrix solution was spiked with international standard proinsulin and international standard insulin or C-peptide for this test.

Cross Reactivity with Insulin

Insulin Conc (pmol/L)	Projected Proinsulin Conc (pmol/L)	Observed Proinsulin Conc (pmol/L)	Recovery (%)
2500	0.00	0.00	100.00
2500	16.09	16.09	100.00
2500	37.50	32.84	87.58

Cross Reactivity with C peptide

Cpeptide Conc (pmol/L)	Projected Proinsulin Conc (pmol/L)	Observed Proinsulin Conc (pmol/L)	Recovery (%)
8250	0.00	0.00	100.00
8250	16.09	16.96	105.45
8250	37.50	31.93	85.15

Table 19. Projected and Observed proinsulin concentrations and percentage recovery when spiked with either insulin or C-peptide, read at 405nm.

Cross Reactivity with Insulin

Insulin Conc (pmol/L)	Projected Proinsulin Conc (pmol/L)	Observed Proinsulin Conc (pmol/L)	Recovery (%)
2500	0.00	0.00	100.00
2500	16.07	16.28	101.32
2500	37.50	32.84	87.58

Cross Reactivity with C peptide

Cpeptide Conc (pmol/L)	Projected Proinsulin Conc (pmol/L)	Observed Proinsulin Conc (pmol/L)	Recovery (%)
8250	0.00	0.00	100.00
8250	16.07	16.65	103.62
8250	37.50	32.11	85.62

Table 20. Projected and Observed proinsulin concentrations and percentage recovery when spiked with either insulin or C-peptide, read at 450nm.

As can be seen by these data the presence of insulin or C-peptide in samples is unlikely to interfere with determination of proinsulin in this assay, with recovery generally as expected.

Freeze/Thaw Test

Pooled serum was spiked with proinsulin international standard and frozen then defrosted 1 to 5 times pre analysis to test the effect of freeze/thaw on measured proinsulin concentration. Baseline measurement of 1 freeze thaw cycle was decided on as this is the most likely scenario of preanalytic sample storage in a clinical laboratory.

Freeze/thaw Plasma & Serum

No. F/T cycles	Plasma Proinsulin Conc (pmol/L)	Recovery Plasma (%)	Serum Proinsulin Conc (pmol/L)	Recovery Serum (%)
1	34.56	100.00	6.57	100.00
2	33.49	96.90	6.89	104.79
3	37.40	108.22	6.21	94.42
4	36.69	106.15	5.95	90.46
5	38.14	110.35	7.34	111.62

Table 2., Proinsulin concentration measured after stated number of freeze/thaw cycles and percentage difference from the baseline point calculated, measured at 405nm.

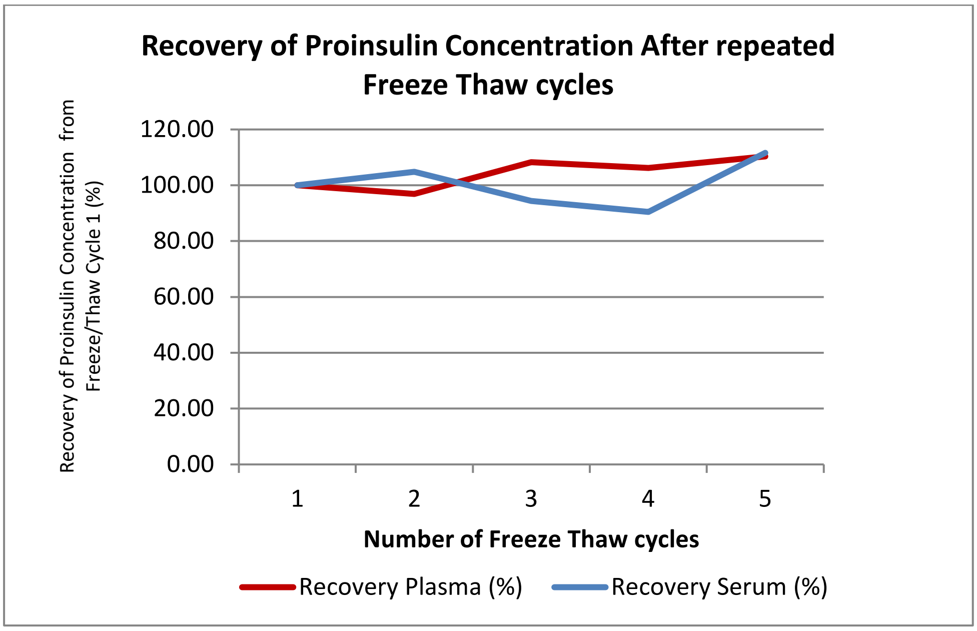

Figure 1. Graph of recovery of proinsulin from baseline after repeated freeze/thaw cycles, measured at 405nm.

Freeze/thaw Plasma & Serum

Number of Freeze Thaw cycles	Plasma Proinsulin Conc (pmol/L)	Recovery Plasma (%)	Serum Proinsulin Conc (pmol/L)	Recovery Serum (%)
1	34.62	0.00	6.73	0.00
2	33.38	-3.57	7.08	5.17
3	37.12	7.21	6.41	-4.70
4	36.90	6.58	6.02	-10.62
5	38.02	9.81	6.55	-2.64

Table 22. Proinsulin concentration measured after stated number of freeze/thaw cycles and percentage difference from the baseline point calculated, measured at 450nm.

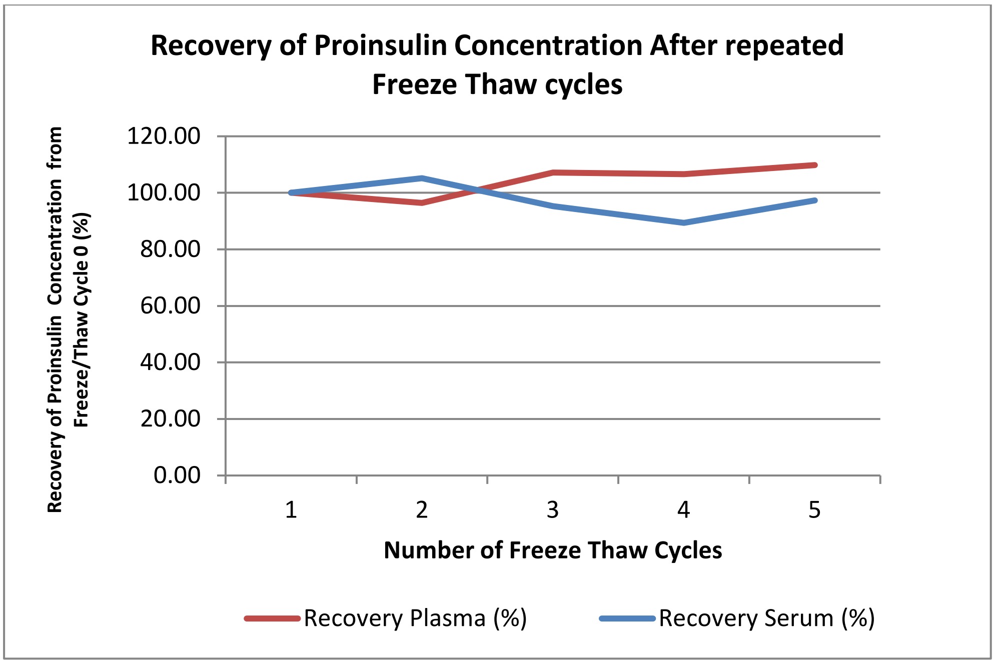

Figure 2. Graph of recovery of proinsulin from baseline after repeated freeze/thaw cycles, measured at 450nm.

The relationship between the freeze thaw cycle and change in the proinsulin concentration measured does not appear to be linear, so drawing conclusions about the effect of repeated freeze/thaw on samples for proinsulin analysis is difficult. In both serum and plasma on two freeze thaw cycles proinsulin concentrations are around or below 5% difference which would be considered acceptable, and account for expected analytical variation. At greater levels of free/thaw cycles the percentage change generally increases to that greater than acceptable, with this difference probably attributable to repeated freeze thaw of the samples.

In looking at these results the previously observed intra assay precision has to also be taken into account. When read at 405nm with a proinsulin concentration of approximately 5pmol/L the coefficient of variation is 7.5%, so the level of error in measurement of proinsulin concentration is already high, so how much of the difference in these values is attributable only to the effect of freeze thaw is difficult to determine.

Linearity

To test the linearity of the assay the calibration points were plotted against the given concentration of proinsulin and Pearson's R correlation coefficient calculated. As standards are assayed in duplicate and the data was taken from 2 plates and average of the 4 values of optical density are used.

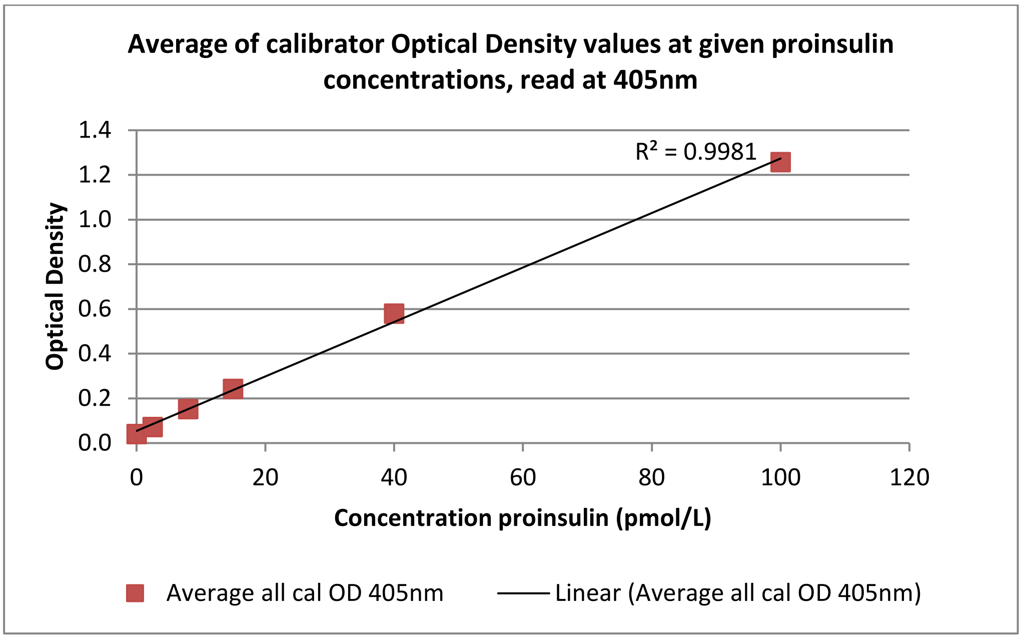

Figure 3. Graph of average optical density at given proinsulin concentration, read at 405nm, with linear trend line and correlation coefficient given.

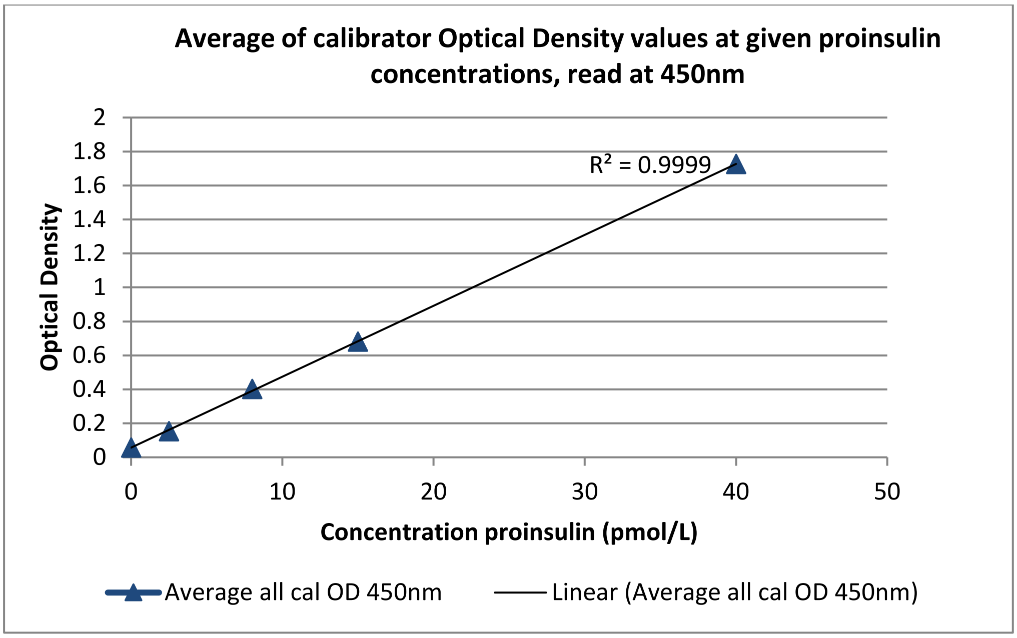

Figure 4. Graph of average optical density at given proinsulin concentration, read at 450nm, with linear trend line and correlation coefficient given.

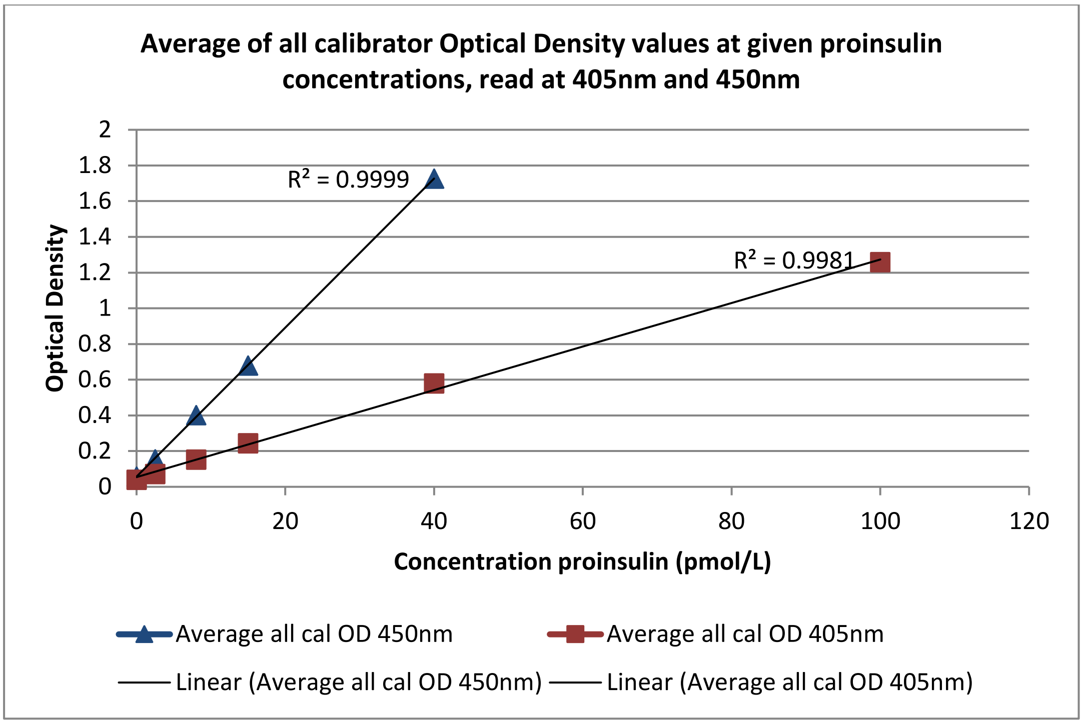

Figure 5. Graph of average optical density at given proinsulin concentration, read at both 405nm and 450nm, with linear trend line and correlation coefficients given.

As can be seen from this data the linearity of the calibrators is very good, with the correlation coefficient at 450nm excellent, R^2=0.9999. When the data from the reads at the two wavelengths are plotted against each other it can be seen that when read at 450nm the range of concentrations is much reduced but as the spread over the optical density is greater; this gives rise to the greater sensitivity at the lower end of the measurement scale.

Interference from Haemolysis

To test for possible interference from haemolysis in samples, a sample spiked with proinsulin international standard had additional solution added at varying levels of haemolysis. The level of haemolysis for each spiked sample was determined by analysing on the Roche (Hitachi) P Module before analysis of proinsulin concentration. Where the haemolysis level equated to zero this was considered the baseline and the percentage difference from this proinsulin concentration calculated.

EFFECT OF HAEMOLYSIS

	HAEMOLYSIS (AU/L)	CONC (pmol/L)	Recovery from HAEM0 (%)
HAEM0	0	28.28	100.00
HAEM1	50	27.94	98.82
HAEM2	100	27.36	96.75
HAEM3	149	30.97	109.52
HAEM4	200	29.03	102.66

Table 23. Proinsulin concentration and percentage difference from haemolysis =0, read at 405nm.

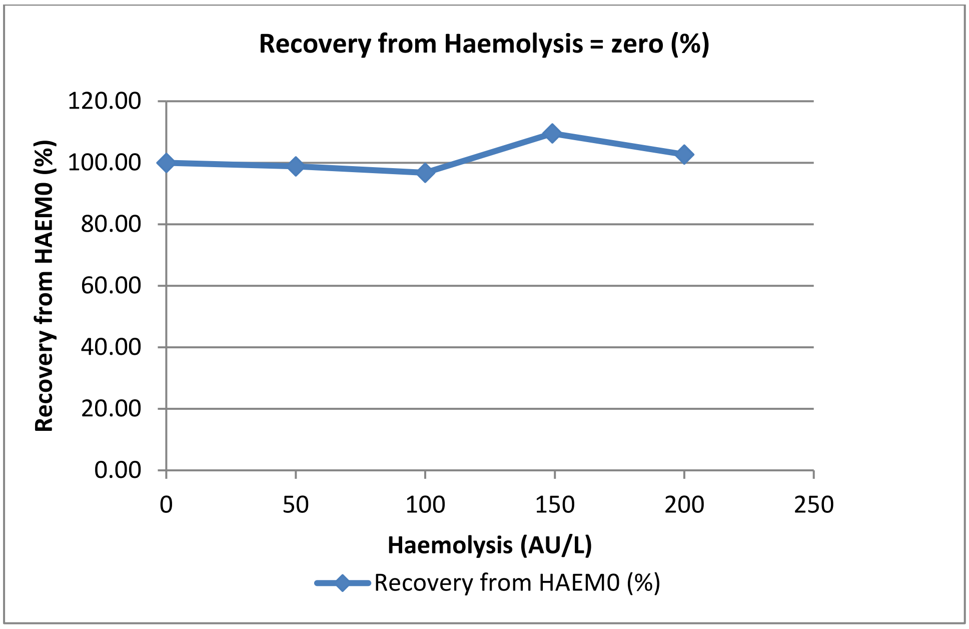

Figure 6. Graph of recovery from haemolysis =0, with increasing levels of haemolysis read at 405nm.

EFFECT OF HAEMOLYSIS

	HAEMOLYSIS (AU/L)	CONC (pmol/L)	Recovery from HAEM0 (%)
HAEM0	0	28.60	100.00
HAEM1	50	28.50	99.65
HAEM2	100	27.81	97.22
HAEM3	149	31.49	110.09
HAEM4	200	29.54	103.26

Table 24. Proinsulin concentration and percentage difference from haemolysis =0, read at 450nm.

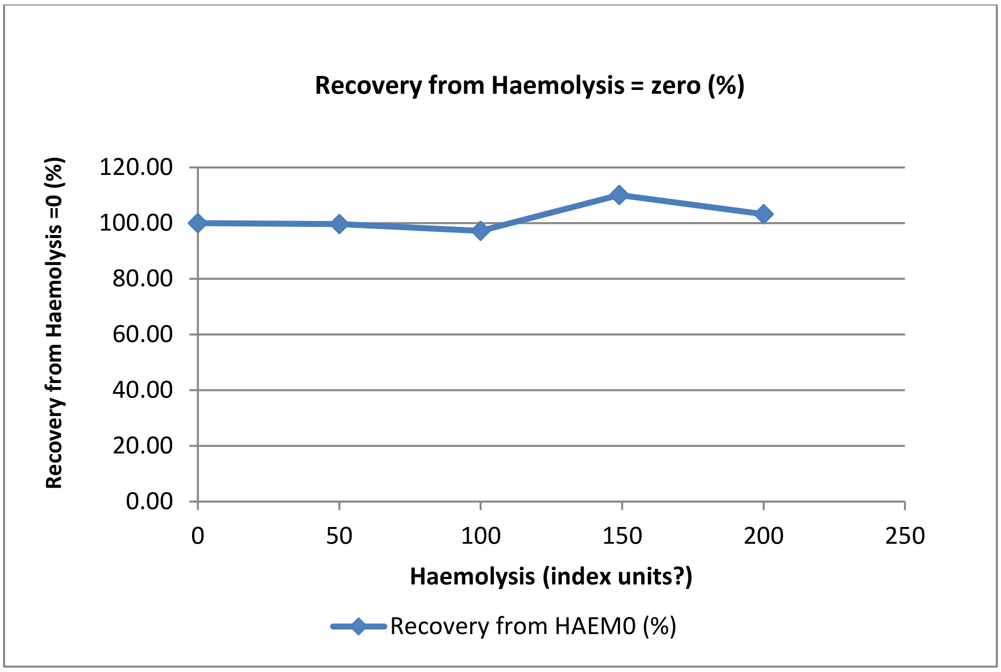

Figure 7. Graph of recovery from haemolysis =0, with increasing levels of haemolysis read at 405nm.

There appears to be no clear relationship between increasing haemolysis and change in measured proinsulin concentration. The point measuring 149 AU/L haemolysis, at 10.1% different from the value measured at haemolysis=0, is still within acceptable levels of analytical variability. This data supports no interference from haemolysis up to a level of 200 AU/L.

Comparison of data obtained at 405nm and 450nm

As the recommendation is to run the assay with 2 reads at 405nm and 450nm a comparison of the obtained proinsulin concentrations has been made to assess the performance of each wavelength read.

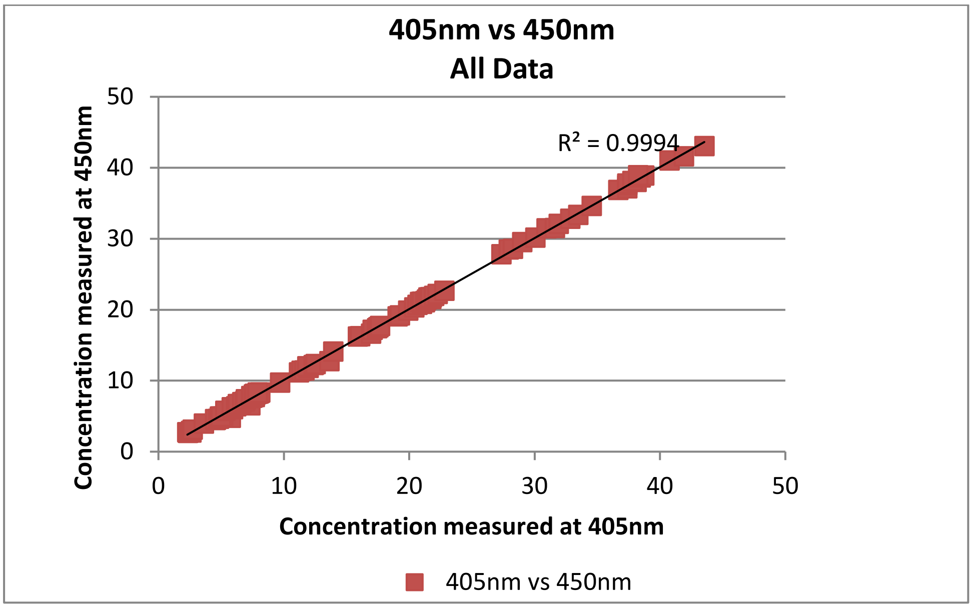

Figure 8. Graph comparing obtained concentrations between readings at 405nm and 450nm, N=102. R^2 value given.

Across the manufacturers range of 2.5pmol/L to 40pmol/L comparable between the two wavelengths there was excellent agreemement in the concentration of proinsulin obtained between the reads at the two wavelengths with an R^2 value of 0.9994.

As the read at 450nm is recommended due to the greater sensitivity at the lower end of the measuring range this comparrison was repeated with only values up to 10pmol/L.

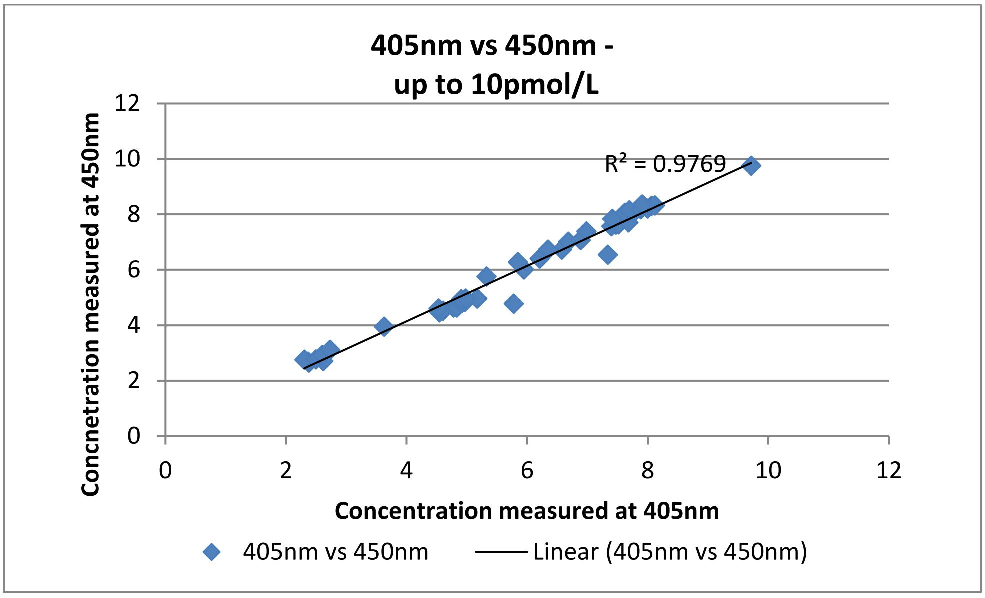

Figure 9. Graph comparing obtained concentrations between readings at 405nm and 450nm, range 2.5pmol/L to 10pmol/L, N=42. R^2 value given.

As can be seen the agreement at the lower end of the measuring range is not as good as generally over the whole range but this is expected with the increased sensitivity at 450nm. Also as has been previously shown with Intraassay and limit of quantification experiments, the precision particularly at 405nm, is not acceptable below 8pmol/L so the likelihood of disagreement on obtained concentration at the two wavelengths is greater.

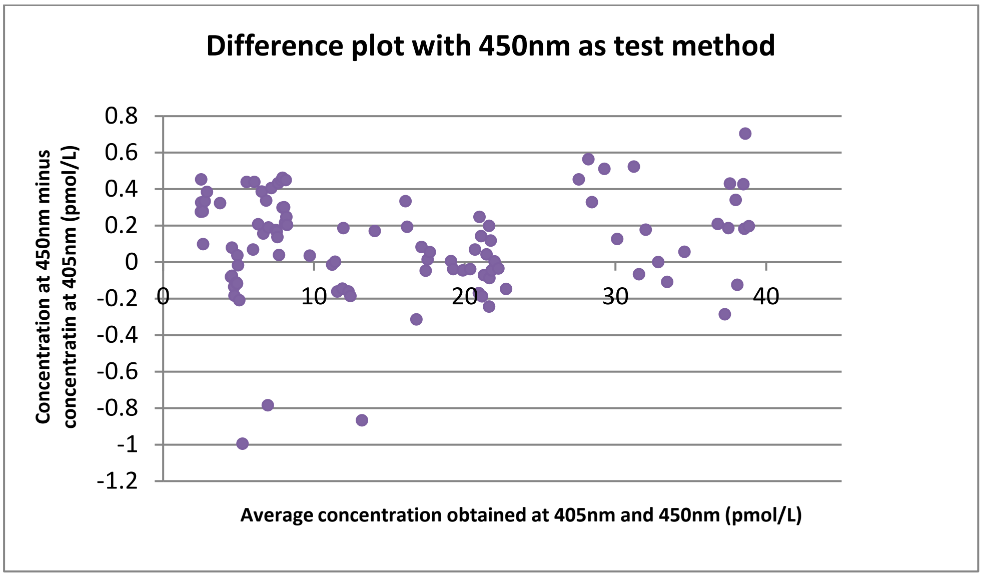

Figure 10. Bland-Altman Difference plot with 450nm as the test method .N=99.

From the above difference plot it can be seen that at 450nm the concentrations obtained tend to be greater than those at 405nm, with several outliers from this. This difference is consistent across the given range of 2.5pmol/L to 40 pmol/L. Relatively though this effect is probably not that great, at the lower end of the measurement scale the precision has been shown to be poorer and at the higher scale a difference of up to 0.6pmol/L is proportionally low.

Conclusions

From the experiments and data analysis performed some conclusions can be drawn in assessing the performance characteristics of the TECO Medical Intact Proinsulin ELISA kit.

- The measuring range of the assay at each wavelength can confidently be defined as;
  - 5 – 40pmol/L measuring range at 450nm
  - 8 – 100pmol/L measuring range at 405nm

  The intraassay precision within these ranges has been shown as good, the limit of detection is acceptable, as is the upper limit of quantification.
- Recovery of both serum and plasma on addition of a known proinsulin concentration is good.
- Dilution of samples with MultiAssay Diluent up to x2 is acceptable.
- There is no interference with insulin up to 2500 pmol/L or C-peptide up to 8250 pmol/l.
- Freeze/Thaw up to 2 cycles is acceptable, and possibly acceptable up to 5 cycles, with no real significant differences observed between serum and plasma samples.
- The assay calibration is linear.
- Samples are unaffected by Haemolysis up to 200AU/L.
- There are no real differences between measurement at 450nm and 405nm although the greater sensitivity at the lower range has shown value in analysing data at both wavelengths to extend the lower reporting range.
